# Deciphering *Cronobacter sakazakii* Pathogenesis: From Host Invasion to Future Directions

**DOI:** 10.3390/microorganisms14030572

**Published:** 2026-03-03

**Authors:** Chen Zhang, Shuyu Liu, Bowen Zhang, Yiqin Chen, Qingli Dong, Peng Lan, Jiancang Zhou, Lei Fang

**Affiliations:** 1 Department of Critical Care Medicine, Sir Run Run Shaw Hospital, College of Medicine, Zhejiang University, Hangzhou 310016, China; 22318487@zju.edu.cn (C.Z.); 3230100925@zju.edu.cn (S.L.); 3230100380@zju.edu.cn (B.Z.); penglan1993@zju.edu.cn (P.L.); 2 Department of Psychology and Human Development, University College London, Faculty of Education and Society, Bloomsbury Campus, London WC1E 6BT, UK; yiqin.chen.22@alumni.ucl.ac.uk; 3 School of Health Science and Engineering, University of Shanghai for Science and Technology, Shanghai 200093, China; qdong@usst.edu.cn

**Keywords:** *Cronobacter sakazakii*, virulence determinants, pathogenesis, foodborne pathogens, powdered infant formula, biocontrol strategies

## Abstract

*Cronobacter sakazakii* is a formidable foodborne pathogen that poses a severe, often fatal threat to neonates and immunocompromised individuals, with contaminated powdered infant formula as the primary transmission vehicle. Infections can lead to devastating conditions, such as meningitis, necrotizing enterocolitis, and sepsis, with alarmingly high mortality rates. Clinical management is hampered by the lack of standardized treatment guidelines and the emergence of antibiotic resistance. However, ongoing research into its molecular pathogenesis continually covers novel targets for intervention. In this review, we synthesize recent advances in our understanding of the sophisticated mechanisms that enable *C. sakazakii* to cause disease. We argue that its virulence hinges on a multi-faceted strategy, including efficient host invasion and tissue penetration via outer membrane proteins, sophisticated immune evasion tactics for intracellular survival, a repertoire of regulated virulence determinants, resilient biofilm formation, and robust stress response systems that ensure environmental persistence. As research continues to decipher these intricate host–pathogen interactions, we highlight promising future directions, including the development of rapid on-site diagnostics, the application of effective biocontrol strategies like phage therapy and probiotics, and the formulation of targeted therapeutic regimens. Ultimately, integrating these multifaceted insights is paramount to developing comprehensive strategies for preventing and controlling the global health burden imposed by *C. sakazakii*.

## 1. Introduction

*Cronobacter sakazakii*, a Gram-negative opportunistic pathogen within the *Enterobacteriaceae* family, poses a serious threat to neonates, preterm infants, and immunocompromised adults. It is associated with severe clinical outcomes, including meningitis, necrotizing enterocolitis (NEC), and sepsis [1], with reported neonatal fatality rates ranging from 40% to 80%. Survivors often suffer from long-term neurological and developmental impairments [2,3,4]. The taxonomy of *C. sakazakii* has undergone several revisions. Formerly classified as *Enterobacter sakazakii*, the organism was reclassified in 2007 into the newly established genus *Cronobacter* by the International Committee on Systematics of Prokaryotes (ICSP) Subcommittee, based on a polyphasic taxonomic approach. The genus now includes seven recognized species: *C. sakazakii*, *C. condimenti*, *C. dublinensis*, *C. malonaticus*, *C. muytjensii*, *C. turicensis*, and *C. universalis* [5,6]. Among them, *C. sakazakii*, *C. malonaticus*, and *C. turicensis* are strongly associated with severe human infections, whereas the others are primarily environmental with limited evidence of clinical significance [7].

*C. sakazakii* occupies a broad ecological niche and has been isolated from various natural, industrial, and healthcare environments. The primary vehicle for neonatal infection is reconstituted powdered infant formula (PIF), with contamination occurring either intrinsically or extrinsically through post-processing handling [8]. The World Health Organization (WHO) recognizes *Cronobacter* and *Salmonella* as the only bacterial contaminants in PIF consistently linked to fatal neonatal infections [9].

In response to strengthened regulatory oversight and improvements in manufacturing practices, the prevalence of *C. sakazakii* contamination in PIF has significantly declined. Surveillance data from 2004 to 2017 demonstrate a marked reduction in *C. sakazakii* detection rate in PIF, from 12.6% to approximately 1–3%, likely due to improved manufacturing and quality control practices [10,11]. Despite these improvements, microbiological safety remains a pressing concern—particularly in regions such as China, where an estimated 85% of neonates rely on formula feeding [12]. Additionally, *C. sakazakii* has been detected in a range of non-dairy food products, including spices (22% to 57%), cereal-based products (approximately 25%), edible fungi (13% to 30%), fruits (2%) and ready-to-eat (RTE) foods [10]. Besides these primary commodities, *Cronobacter* spp. have also been isolated from aquatic foods, including fish and shellfish, further highlighting their broad ecological distribution and potential food safety relevance [13]. Environmental reservoirs are similarly diverse. The organism has been recovered from water, dust, soil, plant material, and insects and rodents, indicating an ability to persist across many habitats [14]. Some studies have shown the isolation of *C. sakazakii* from human and animal feces, but fecal positivity is more often a consequence of foodborne infection rather than a primary source of food contamination [15]. As a result, the International Commission for Microbiological Specifications for Foods (ICMSF) classifies *C. sakazakii* as a severe hazard for vulnerable populations, capable of causing life-threatening infections, substantial chronic sequelae, prolonged illnesses, and serious public health implications [16,17].

**Figure 1:**
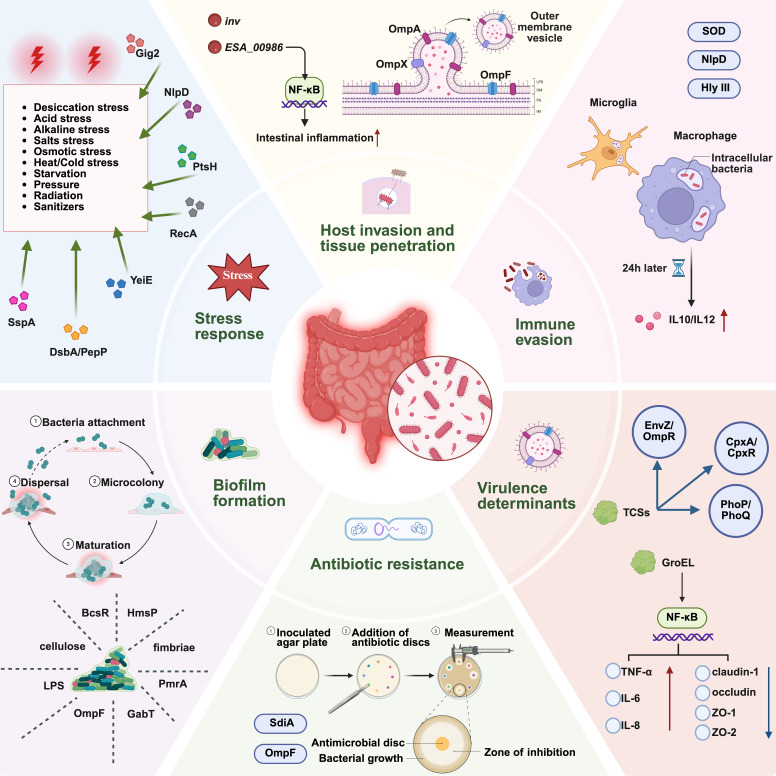
Pathogenic mechanisms of *C. sakazakii*. Schematic overview of major factors contributing to *C. sakazakii* pathogenesis, including stress response systems (e.g., NlpD, RecA, PtsH), biofilm formation, host invasion and tissue penetration mediated by outer membrane proteins and vesicles, immune evasion strategies, virulence regulation by two-component systems (e.g., EnvZ/OmpR, CpxA/CpxR, PhoP/PhoQ), and antibiotic resistance assessed via disc diffusion assays. Created in BioRender. Zhang, C. (2026) https://BioRender.com/gxinm6w (accessed on 21 February 2026).

This pathogen harbors a wide array of virulence factors that enable epithelial adhesion, invasion of host cells, and cellular injury, which underlie the catastrophic outcomes in neonates (Figure 1). These virulence mechanisms remain under active investigation. This review summarizes the current understanding of the clinical features, epidemiology, pathogenesis, diagnosis, and treatment strategies of *C. sakazakii* infections, with particular emphasis on key virulence determinants and their roles in host–pathogen interactions, thereby providing a comprehensive perspective on its public health significance.

## 2. Symptoms, At-Risk Populations, and Important Outbreaks

*C. sakazakii* exhibits a marked predilection for infecting neonates and infants, particularly those who are immunocompromised and consume PIF. This susceptibility is attributed to multiple host-related factors, including reduced gastric acidity (pH < 4), insufficient immunity, immature intestinal barriers, and an underdeveloped gut microbiome [18]—all of which enhance bacterial survival during gastric passage and promote intestinal colonization. These vulnerabilities synergistically heighten susceptibility to severe clinical outcomes such as NEC, meningitis, and sepsis [18]. The core features of NEC, characterized by intestinal necrosis and pneumatosis intestinalis [19], present with a spectrum of symptoms ranging from abdominal distention and bilious vomiting to hematochezia, intestinal perforation, peritonitis, and shock [20]. In 1994, a large-scale outbreak of *C. sakazakii* occurred in a neonatal intensive care unit in France, infecting multiple newborns and causing three deaths, two of whom succumbed to NEC [21].

In adults, sepsis is defined as a dysregulated host response to infection resulting in life-threatening organ dysfunction [22], but a universally accepted definition in infants and neonates is lacking, complicating diagnosis, research, and healthcare management [23]. Due to the often non-specific signs of neonatal sepsis [23], such as apnea, bradycardia, lethargy, temperature instability, peritonitis, and shock, it frequently overlaps with those of NEC in clinical practice [20], leading to diagnostic ambiguity.

*C. sakazakii* also exhibits a strong propensity for invading the central nervous system (CNS). This pathogen can cause fatal meningitis in neonates, particularly preterm infants. Acute-phase manifestations commonly include poor feeding, irritability, seizures, and fever [24]. Survivors frequently experience long-term neurological sequelae, which may include neurodevelopmental delays, brain abscesses, hydrocephalus, intraventricular compartmentalization, and intracerebral hemorrhagic or non-hemorrhagic infarctions, which may progress to cystic encephalomalacia and lifelong disabilities [25]. Another major outbreak occurred in Tennessee in 2001, which documented the first U.S. case of *C. sakazakii* infection linked to PIF [26]. The affected preterm infant (33.5 weeks’ gestation, 1270 g) developed fever, tachycardia, decreased vascular perfusion, and neurologic abnormalities at 11 days after birth. Cerebrospinal fluid (CSF) analysis revealed elevated white blood cell count (32/mm^3^), markedly increased protein (292 mg/dL), and critically low glucose (1 mg/dL), confirming *C. sakazakii* meningitis. Despite antimicrobial treatment, the infant died nine days later due to progressive neurological deterioration. In contrast, *C. sakazakii* infections in adults are uncommon and primarily occur as opportunistic infections in immunocompromised populations [27]. Reported presentations include conjunctivitis, bacteremia (of biliary or urinary origin), wound infections, or pneumonia [1], although disease severity is less pronounced compared to infantile cases [28].

## 3. Pathogenesis

### 3.1. Host Invasion and Tissue Penetration

*C. sakazakii* employs sophisticated mechanisms to breach host barriers. It can adhere to and invade human intestinal epithelial cells, a process critically mediated by outer membrane protein A (OmpA) [29,30,31]. OmpA facilitates invasion across multiple mammalian cell types and contributes to the pathogen’s resistance to the bactericidal activity of blood and serum in neonatal rats [29,32,33]. Functionally, OmpA binds to fibronectin—a major component of the extracellular matrix and cerebral microvascular basal lamina—which likely acts as a transient, non-specific bridge initiating bacterial attachment. The invasion of intestinal epithelial (INT407) cells further requires coordinated participation of both microfilaments and microtubules, with fibronectin serving as the initial mediator between the bacterium and host cells [29].

This pathogen can also traverse the blood–brain barrier (BBB) [34,35], a crucial step in the pathogenesis of meningitis. During invasion of human brain microvascular endothelial cells, *C. sakazakii* differs from *Escherichia coli* (*E. coli*) K1 in that its OmpA-mediated mechanism relies on microtubule reorganization rather than microfilament condensation, and this process is closely associated with activation of host phosphoinositide 3-kinase and PKC-α [32]. Notably, OmpA-mediated invasion of brain endothelial cells occurs independently of fibronectin binding, suggesting that other host receptors or signaling pathways are involved.

OmpX shares the same β-sheet topology as the structurally related OmpA [31]. Research findings indicate that the expression of OmpA and OmpX in *C. sakazakii* is essential for both apical and basolateral adhesion to and invasion of mammalian host cells. The synergistic contribution of these two outer membrane proteins (OMPs) to host cell invasion implies their potential engagement with distinct cellular receptors [31]. Both OmpA and OmpX are incorporated into outer membrane vesicles (OMVs) and are expressed during the stationary phase in *C. sakazakii*, *C. malonaticus,* and *C. turicensis* [36]. Notably, their expression levels are more abundant in virulent *C. sakazakii* strains than in avirulent strains [37]. In addition to OmpA and OmpX proteins, *C. sakazakii* secretes OMVs that are internalized by Caco-2 cells, where they stimulate proliferation and pro-inflammatory responses. These vesicles likely contribute to cytopathogenicity and host cell responses in human intestinal epithelial cells [38].

Bioinformatic analysis has also identified *inv* as an outer-membrane-localized potential virulence factor. Chandrapala et al. [39] confirmed that the putative Inv protein plays a critical role in facilitating *C. sakazakii* ATCC 29544 invasion of mammalian epithelial cell lines (Caco-2, INT-407, and Hep-2), and is necessary for basolateral penetration of Caco-2 cells and bacterial spread in a rat pup model. Furthermore, synergistic interactions between OmpA and Inv were observed in Caco-2 cells and animal models. Another virulence gene, *ompF*, enhances adhesion and invasion of HCT-8 cells, while the *ESA_00986* gene promotes adhesion, invasion, motility, and in vivo colonization, highlighting their positive contributions to virulence [40,41]. *ESA_00986* encodes a virulence factor with an immunoglobulin-like (Ig-like) domain. Studies by Fan et al. [40] demonstrated that deletion of this gene significantly reduced epithelial cell invasion and dissemination into rat tissue. Mechanistically, *ESA_00986* may upregulate intestinal inflammation via the NF-κB signaling pathway, disrupt the intestinal barrier by suppressing the expression of related genes and enhancing biofilm synthesis, and play an active role in bacterial adhesion/invasion processes.

The adhesion and invasion mediated by OmpA, OmpX, and Inv not only explain the efficient colonization of the immature neonatal gut by this bacterium but also constitute the initial molecular basis for triggering NEC and subsequent bacteremia [42]. The ability of OmpA to specifically traverse the BBB mechanistically partly elucidates why *C. sakazakii* is prone to cause severe neonatal meningitis [43].

### 3.2. Immune Evasion

The persistence of certain strains in the neonatal gastrointestinal tract may be related to their *sod* genes, which encode superoxide dismutases (SODs). These enzymes help the bacteria survive under acidic conditions and macrophage-induced oxidative stress by neutralizing reactive oxygen species. Pandemic-associated *C. sakazakii* isolates from neonatal intensive care units were shown to survive in U937 macrophages for up to 48 h [44], and some strains exhibit enhanced intracellular persistence over a 72 h period [45]. Data from Almajed and Forsythe [46] further provided evidence that clinical isolates of this pathogen can survive and proliferate within human microglial cells, and undergo paracellular translocation across Caco-2 and HBMEC cell lines, thus offering a cellular basis for CNS invasion.

Immune modulation also contributes to *C. sakazakii* persistence, as infected macrophages display an increased interleukin (IL)-10/IL-12 ratio after 24 h, suggesting a potential skew toward a type 2 immune response that reduces clearance efficiency [47]. Moreover, a novel counterselection method using gentamicin and acid approach identified the *nlpD* gene as a key acid tolerance factor, maintaining membrane integrity under acid stress and conferring resistance to macrophage-mediated killing [48]. Hemolysin III (Hly III, encoded by the gene *ESA_00432*) plays a critical role in neuroinvasion and BBB translocation. It is noteworthy, however, that its expression incurs a fitness cost under non-invasive conditions, reflecting the complex trade-offs in the survival strategies of *C. sakazakii* across different environments [49].

The prolonged intracellular survival within macrophages and microglial cells allows *C. sakazakii* to evade routine antibiotic killing and establish latent infections, providing a biological explanation for clinical relapse and treatment failure [50,51]. The skewing towards a Th2-type immune response may further impair the already compromised cellular immunity in preterm infants, leading to inefficient pathogen clearance and protracted illness [52].

### 3.3. Virulence Determinants

*C. sakazakii* employs a synergistic action of multiple virulence factors and regulatory systems to achieve host cell invasion, stress survival, and immune evasion, which ultimately leads to severe clinical outcomes. In terms of regulatory systems, two-component systems (TCSs) mediate bacterial adaptation to gastrointestinal stress and metabolic cues [53]. Specifically, EnvZ/OmpR, PhoP/PhoQ, and CpxA/CpxR play central roles in regulating stress responses to desiccation, oxidative stress, and other environmental challenges. Their absence leads to reduced bacterial adhesion and invasion, diminished colonization in vivo, and attenuated virulence [54,55]. Functional studies highlight their importance. One study constructed *ΔphoPQ* knockout and complementary strains to evaluate the role of the PhoP/PhoQ system in *C. sakazakii* ATCC BAA-894 under various environmental stresses including low magnesium, acidic pH, polymyxin B, heat, osmotic pressure, oxidation, and bile salts. The experiments revealed that the survival ability of the *ΔphoPQ* strain was significantly reduced under these multiple stress conditions, while the complementary strain partially restored tolerance [56]. Recent research further demonstrated that knockout of PhoP/PhoQ significantly reduced the survival of *C. sakazakii* in the mouse small intestine and alleviated inflammation by modulating the TLR4/NF-κB pathway, indicating a dual role in stress adaptation and immune activation [57]. Similarly, the expression of *nlpD* is regulated by the environmental stress system EnvZ/OmpR. Under acidic conditions, EnvZ activates the transcription of *nlpD*, thereby enhancing bacterial acid resistance. Moreover, compared to the wild-type strain, the *nlpD* mutant exhibited attenuated virulence in a rat model [48]. The TCS component *kdpE* gene also contributes to virulence. Deletion of the *kdpE* gene attenuated virulence and improved rat survival by reducing bacterial growth, but paradoxically enhanced desiccation tolerance while impairing biofilm formation due to GrpE upregulation [58].

Through bioinformatics analysis and PCR detection, Cruz et al. [59] identified three hypothetical virulence genes: the siderophore interaction protein (*sip*), type III hemolysin (*hly*), and plasminogen activator gene (*cpa*). Franco et al. [60] further investigated the role of the outer membrane protease Cpa in serum resistance of *C. sakazakii*. Cpa was found to significantly enhance serum resistance by degrading complement components (C3, C3a, and C4b) and activating plasminogen, suggesting that it represents a key virulence factor contributing to the pathogenic potential of *C. sakazakii*. Additionally, the RNA chaperone Hfq was shown to promote invasion and intracellular survival of *C. sakazakii* in host cells by regulating bacterial small RNAs [61], whereas the transcriptional regulator CklR enhanced oxidative stress resistance by activating the *suf* operon, thereby contributing to sepsis and meningitis [62].

Other important virulence- and fitness-associated factors have also been identified. Labp, which directly binds to LpxA, significantly enhances its catalytic activity in lipopolysaccharide biosynthesis. A mutant lacking CSK29544_02616 (referred to as *labp*) showed significantly reduced ability to invade epithelial cells and to be phagocytosed by macrophages, along with notable changes in OMP composition, lipopolysaccharide content, and phospholipid production distribution [63]. The flagellar hook-associated protein gene *flgK* encodes a main component of the flagellar complex. It facilitates flagellum assembly and function, contributing to motility and host cell invasion [64]. *pdxY* (encoding pyridoxal kinase) plays a key role in vitamin B_6_ metabolism by catalyzing the phosphorylation of pyridoxal to generate pyridoxal 5′-phosphate (PLP)—an essential cofactor for numerous enzymatic reactions involved in amino acid metabolism and bacterial growth, thereby affecting virulence and growth [65]. Molecular chaperones GroEL and DnaK play key roles in stress response and virulence maintenance. Among them, recombinant GroEL protein can activate the NF-κB signaling pathway, leading to the release of more pro-inflammatory cytokines (TNF-α, IL-6, and IL-8) and the downregulation of tight junction proteins (claudin-1, occludin, ZO-1, and ZO-2), which collectively result in a dose-dependent toxic effect on host cells. Moreover, DnaK and GroEL exhibit partial functional complementarity, and the upregulation of GroEL expression may serve as a compensatory mechanism for DnaK deficiency [66,67]. The *rpfB* gene, which encodes a long-chain acyl-CoA synthase, plays a crucial role in fatty acid metabolism in *C. sakazakii*. Deletion of *rpfB* significantly reduced bacterial growth and virulence in a mouse infection model, revealing its regulatory role in coordinating metabolic networks with pathogenicity [68].

Collectively, these virulence determinants and regulatory systems provide a mechanistic explanation for the severe clinical outcomes observed in neonatal *C. sakazakii* infections. By enhancing the bacterium’s ability to survive gastrointestinal stress, invade epithelial and endothelial barriers, resist complement-mediated killing, and trigger excessive inflammatory signaling, these factors directly contribute to the rapid progression from intestinal colonization to necrotizing enterocolitis, bacteremia, and CNS invasion. The capacity of *C. sakazakii* to modulate host immunity, persist intracellularly, and withstand harsh environmental conditions further underlies the high mortality and long-term neurological sequelae characteristic of neonatal disease.

### 3.4. Biofilm Formation

Bacteria attach to moist surfaces and form multicellular, surface-adherent biofilms, which are considered major contributors to infectious diseases and are often associated with persistent antibiotic resistance [69,70,71]. The survival capability of *C. sakazakii* in low-moisture foods, such as dehydrated infant formula, is closely associated with its ability to form biofilms, which serve as a critical strategy for coping with adverse environmental conditions. The pathogen forms sessile biofilms on both biotic and abiotic surfaces, facilitated by a polyanionic extracellular polysaccharide (ESP) that enables stronger attachment to abiotic surfaces [72].

Multiple studies have elucidated the molecular regulatory network underlying biofilm formation. For instance, Hartmann et al. [73] constructed a random transposon mutant library through which they screened, analyzed, and identified a series of genes related to biofilm formation. The results indicated that cellulose and fimbriae, as well as two novel candidates (*ESA_00281* and *ESA_00282*), play important roles in the biofilm formation of *C. sakazakii*.

Further work by Wang et al. [74] indicated that alterations in lipopolysaccharide (LPS) structure—such as the knockout of the heptosyltransferase I gene *ESA_04107*, which produces only Kdo_2_-lipid A without O-antigen—led to impaired growth, increased membrane permeability, enhanced surface hydrophobicity, and promoted auto-aggregation and biofilm development. The cellulose synthesis-related gene *bcsR* has also been confirmed to participate in this process; its deletion exhibited an approximately 50% reduction in biofilm formation, accompanied by decreased levels of carotenoids, fatty acids, and amides but elevated cellulose content. *bcsR* thus acts as a negative regulator of cellulose synthesis but positively modulates biofilm formation and cellular adhesion/invasion [75]. Furthermore, *ompF* influences biofilm formation through LPS regulation [41], and the response regulator *pmrA* contributes by modulating motility and the expression of biofilm-associated genes [76]. More recently, studies have shown that HmsP and cyclic-di-GMP signaling are involved in biofilm formation. Comparative proteomic analysis identified the lysozyme inhibitor protein LprI as a potential key factor—it was downregulated in an *hmsP* mutant and upregulated in a cyclic-di-GMP-deficient strain. Further investigation of the *lprI* knockout strain revealed significantly weakened biofilm formation and reduced virulence in a rat infection model [77]. γ-Aminobutyric acid (GABA) also plays a documented role in osmoprotection and biofilm regulation [78]. Its metabolism involves both synthesis (via glutamate decarboxylase, Gad) and degradation (via GABA aminotransferase, encoded by *gabT*) [79,80]. Disruption of the degradation pathway, as demonstrated by *gabT* knockout, leads to GABA accumulation, which in turn enhances biofilm formation and stress resistance under hypotonic and acidic conditions. The protective effects are mechanistically linked to GABA’s function as an osmoprotectant and its role in consuming protons during acid stress [81]. Thus, the *gab* gene cluster, particularly its catabolic branch, critically influences GABA homeostasis and stress adaptation.

These biofilm-associated determinants provide a mechanistic basis for the remarkable environmental persistence and clinical severity of *C. sakazakii* infections. Biofilm formation enhances survival in low-moisture foods such as PIF, facilitates resistance to desiccation, disinfectants, and host immune defenses, and promotes prolonged contamination of food-processing environments [82].

### 3.5. Stress Response

Studies have shown that various stress-related proteins play critical roles in the environmental adaptation of this bacterium. *C. sakazakii* exhibits remarkable desiccation tolerance and can survive for prolonged periods under extremely low water activity conditions. This phenotype is supported by a set of specialized physiological and genetic mechanisms. Physiologically, the organism accumulates compatible solutes such as trehalose, which stabilize cellular membranes and protect macromolecules from dehydration-induced damage [83]. At the genetic level, to systematically elucidate the metabolic basis of desiccation tolerance, integrated metabolomic and transcriptomic analyses indicated that desiccation-resistant strain *C. sakazakii* CS 34 upregulated betaine synthesis and transport after desiccation treatment. Through the construction of *betA*, *betB*, and double knockout mutants, it was confirmed that the deletion of these genes resulted in decreased desiccation survival rates, increased cellular structural damage, greater content leakage, accumulation of choline, and reduction in betaine, demonstrating the critical role of the betaine synthesis pathway in resisting desiccation [84]. Furthermore, functional screening and proteomic analysis identified Gig2, a DUF1479-family oxidase, as a novel key factor in desiccation stress. Its deletion caused a significant reduction in desiccation survival without affecting surface hydrophilicity or normal growth, while molecular docking suggested that phloretin binds to Gig2 with high affinity, impairing desiccation tolerance and highlighting Gig2 as a potential control target in low-moisture foods [85]. Deletion of *nlpD* also led to reduced desiccation tolerance, diminished biofilm formation, and altered surface properties, highlighting its function in environmental adaptation [86]. These mechanisms underpin the organism’s ability to persist in PIF and other low-moisture environments, contributing to its epidemiological significance in foodborne neonatal infections.

On the other hand, genes associated with bacterial envelope biogenesis and stability—such as *dsbA* (encoding a disulfide bond oxidoreductase), which catalyzes proper disulfide bond formation in periplasmic proteins, and *pepP* (encoding an aminopeptidase), which degrades peptides and contributes to protein turnover—have been confirmed to significantly regulate heat resistance, desiccation tolerance, and various stress responses by maintaining envelope integrity. They also influence biofilm formation, motility, host cell adhesion, invasion, and intracellular survival [87]. The RecA protein, a central factor in DNA repair and recombination, was found to modulate both bacterial resilience and virulence. Knockout of *recA* resulted in growth defects, reduced desiccation tolerance, and impaired biofilm formation, thereby diminishing environmental adaptability and pathogenicity [88].

The ability of *C. sakazakii* to persist in extreme environments, such as desiccation and acidic conditions, is strongly linked to its diverse stress-response mechanisms. Studies have shown that various stress-related proteins play critical roles in the environmental adaptation of this bacterium. For instance, Zhan et al. [89] found that a homolog of the *E. coli* regulatory factor SspA plays an important role in the stress response of *C. sakazakii* strain BAA-894. Deletion of *sspA* resulted in significantly reduced acid tolerance, while its desiccation tolerance varied with the growth medium, being enhanced in Luria–Bertani broth but reduced in M9 medium. YeiE, a LysR-type transcriptional regulator, recognizes ligands via its DNA-binding and regulatory domains. Subsequent research confirmed that sulfite serves as its physiological ligand, and YeiE assists the bacterium in resisting sulfite toxicity [90]. The *ptsH* gene (encoding the phosphocarrier protein HPr) is also a core component of the phosphoenolpyruvate-dependent phosphotransferase system (PTS). By mediating sugar uptake and phosphorylation, it links nutrient availability to stress adaptation, influencing tolerance to high temperature, gastric fluid, oxidative stress, and osmotic stress [91].

The remarkable ability of *C. sakazakii* to persist in food processing environments—particularly in low-moisture food matrices such as PIF—represents a critical food safety challenge [92]. *Cronobacter* exhibits exceptional desiccation tolerance, capable of surviving for months to years in dehydrated foods and on dry surfaces commonly encountered in manufacturing facilities [93]. In addition to desiccation, *Cronobacter* demonstrates considerable resistance to osmotic stress, acidic conditions, and thermal stress [83]. It is worth noting that sublethal pressures encountered during food processing can induce cross-protective effects. For example, early dehydration treatment can enhance subsequent heat resistance, which further exacerbates the difficulty of eradication work [18]. The ability to form biofilms on abiotic surfaces (stainless steel, silicone, polycarbonate) commonly used in food production equipment provides an additional survival reservoir and contributes to persistent contamination and post-processing recontamination events [94]. Overall, the diverse stress-response systems of *C. sakazakii* enable the bacterium to survive the harsh conditions encountered from PIF to the neonatal gut. By conferring resistance to acidity, bile salts, heat, desiccation, oxidative stress, and envelope damage, these pathways allow the organism to persist in low-moisture foods, withstand gastric passage, and maintain viability during early intestinal colonization. The tight coupling between stress adaptation and virulence enhances adhesion, invasion, and immune evasion, thereby facilitating progression to necrotizing enterocolitis, bacteremia, and CNS infection. Understanding these adaptive strategies is essential for developing effective control measures, including hygienic design, environmental monitoring, and process-based interventions such as optimized dry cleaning and thermal inactivation protocols.

### 3.6. Antibiotic Resistance

Several virulence determinants also participate in antibiotic resistance, demonstrating the overlap between pathogenicity and persistence. For example, OmpF is not only involved in biofilm regulation—its absence also significantly enhances bacterial resistance to multiple antibiotics, such as ampicillin, tetracycline, and ciprofloxacin. This finding suggests its dual role as a key porin for the passive transport of antibiotics and a negative regulator of resistance [41]. Likewise, the quorum-sensing regulator *sdiA* enhances antibiotic resistance, as its deletion renders *C. sakazakii* more sensitive to multiple antibiotics, with kanamycin showing the most pronounced bacteriostatic effect [95].

Beyond the specific virulence–resistance overlap described above, *Cronobacter* isolates from clinical, food, and environmental sources have exhibited resistance to a broad spectrum of clinically important antibiotics. The historical evolution of treatment strategies reflects the dynamic nature of this resistance. Before the mid-1980s, *Cronobacter* infections were typically treated with ampicillin, gentamicin, and/or chloramphenicol [96]. Willis and Robinson specifically recommended the combination of gentamicin and ampicillin for meningitis caused by this pathogen [97].

However, subsequent surveillance revealed a marked shift in susceptibility patterns. Lai [27] reported that *Cronobacter* isolates exhibited widespread resistance to ampicillin, cephazolin, and broad-spectrum penicillins, while remaining uniformly sensitive to trimethoprim/sulfamethoxazole and aminoglycosides. The emergence of extended-spectrum β-lactamase (ESBL) activity was documented by Caubilla-Barron et al. in two isolates [98], and food-derived strains resistant to cephalosporins or ampicillin—yet still susceptible to tetracycline—have also been described [30]. Al-Nabulsi et al. demonstrated that streptomycin, gentamicin, kanamycin, and ciprofloxacin remained effective against *C. sakazakii* under both stressed and non-stressed conditions, suggesting these agents as suitable therapeutic options [99]. Interestingly, Hochel et al. observed strain-dependent variability in tetracycline sensitivity among different *Cronobacter* isolates [100].

Although comprehensive surveillance data are limited, several studies have documented resistance of *Cronobacter* spp. to β-lactams, cephalosporins, tetracyclines, and aminoglycosides [101]. Many resistance traits are plasmid-borne or linked to mobile genetic elements, facilitating horizontal gene transfer and contributing to the emergence of multidrug-resistant strains [102,103]. *C. sakazakii* 505108, isolated from a neonate with severe pneumonia, harbors three resistance plasmids belonging to the IncHI2, IncX3, and IncFIB incompatibility groups, which carry an extensive array of antibiotic resistance genes, including those for carbapenems, aminoglycosides, tetracyclines, phenicols, and sulphonamide/trimethoprim, mobilized by insertion sequences, integrons, and transposons [101]. These resistance patterns not only complicate clinical treatment but also enhance environmental persistence and survival under processing-related stresses.

The growing resistance of *Cronobacter* spp. to clinically important antibiotics is increasingly attributable to the acquisition of specific, horizontally acquired resistance genes. Whole-genome sequencing (WGS) has now pinpointed a range of definitive resistance determinants in isolates from diverse sources. For β-lactams, in addition to the chromosomally encoded intrinsic β-lactamase gene *blaCSA*, plasmid-borne acquired β-lactamase genes such as *blaTEM-1* have been repeatedly identified in food and clinical isolates. Sulphonamide and trimethoprim resistance are conferred by genes including *sul1*, and *dfrA12*, while tetracycline resistance is associated with *tet(A)* and chloramphenicol resistance with *floR*—all of which have been documented in *C. sakazakii* [104,105]. *C. sakazakii* is known to have an open pangenome, indicating considerable accessory gene diversity across strains. However, how this genomic variation relates to its alternative lifestyles, such as adaptation to various food types versus persistence on processing facility surfaces, remains unclear [106]. Another study by Zeng et al. [107] provided the first evidence that the silent spread of *mcr-9.1* in *C. sakazakii* ST13 and ST256 isolates was linked to IncFIB and IncHI2 plasmids, respectively. The IncHI2 plasmid carrying *mcr-9.1* was shown to be transferable from the ST256 strain, indicating substantial potential for dissemination among clinically relevant bacteria. The identification of genetic determinants associated with the persistence of *C. sakazakii* across different food system stages and geographic regions could contribute to the advancement of genotype-based risk assessment strategies.

## 4. Diagnosis

The isolation and identification of *C. sakazakii* are critical for accurate diagnosis of infections, particularly in neonates where delayed treatment can have severe consequences. The methods available for these purposes are summarized in Table 1 and Table 2.

### 4.1. Detection of C. sakazakii

Given the severe clinical implications of *C. sakazakii* infection, rapid and sensitive detection of the pathogen directly from suspicious samples is essential for early diagnosis and effective infection control. Three primary approaches are currently employed to detect *C. sakazakii* in food or clinical specimens: conventional culture-based methods, immunological assays, and molecular biology-based techniques [117].

Traditional detection methods are used to isolate and detect *C. sakazakii* in milk and dairy products according to ISO/TS 22964. The procedure includes: (i) pre-enriching in buffered peptone water at 37 ± 1 °C for 16–20 h; (ii) selectively enriching by inoculating the culture into modified lauryl sulfate tryptose (mLST) broth supplemented with vancomycin and incubating at 44 °C for 24 h; (iii) plating onto chromogenic *C. sakazakii* agar and incubating at 44 °C for 24 h; and (iv) isolating and purifying of presumptive colonies on *Cronobacter* chromogenic medium [108]. This approach is both time- and labor-intensive, typically requiring approximately 5–7 days to yield a positive result, delaying early intervention and exhibiting poor sensitivity for heat-injured or viable but non-culturable cells (VBNC) [118,119]. The VBNC state is a survival strategy adopted by many bacteria, including *Cronobacter* spp., in response to environmental stresses such as nutrient starvation, desiccation, or temperature fluctuations [120]. In this state, cells are metabolically active and remain viable, but lose their ability to grow on routine culture media, making them undetectable by conventional plate count methods [121]. Crucially, *C. sakazakii* has been documented to enter the VBNC state, particularly in low-moisture foods like PIF [122]. These VBNCs pose a significant food safety risk, as they may resuscitate and regain culturability upon encountering favorable conditions, thereby retaining their pathogenic potential [123].

Immunological detection refers to enzyme-linked immunoassay techniques that rely on the specific interaction between antibodies and antigens, such as the enzyme-linked immunosorbent assay (ELISA), delivering faster and more cost-effective detection of *C. sakazakii* specific antibodies or antigens than conventional culture [117,124,125]. While ELISA demonstrates high specificity for macromolecular antigens, it requires 4–6 h to complete, shows limited sensitivity (≈10^4^ CFU/mL vs. ≈10^1^ CFU/mL for PCR), and cannot distinguish VBNCs [125,126].

Over the past decade, detection technologies for *C. sakazakii* have shifted from time-consuming cultivation-based methods to rapid molecular and analytical techniques, greatly improving sensitivity and throughput. Among nucleic acid-based assays, polymerase chain reaction (PCR) and its derivatives remain the cornerstone for analyzing foodborne pathogens and food components due to their high sensitivity and strong specificity [127,128]. Zimmermann et al. [129] developed a fast real-time PCR (qPCR) assay targeting *ompA* that achieved a detection limit as low as 0.01 CFU/g within 24 h. These PCR-based assays are effective; however, their reliance on costly thermocyclers and specialized laboratory settings limits their broader application for rapid on-site detection at the grassroots level [126,130,131]. Loop-mediated isothermal amplification (LAMP) offers a low-cost and instrument-free alternative, enabling visual detection of *C. sakazakii* in PIF within 1 h [132,133,134].

To overcome the limitation of VBNC detection, several molecular methods have been developed. Propidium monoazide (PMA)-qPCR selectively detects viable cells: PMA is a DNA intercalating dye that can penetrate dead cells with compromised membranes and intercalates into their DNA, thereby preventing PCR amplification and excluding signals from dead cells [135]. PMAxx is a new improved version of PMA that is more effective in eliminating dead cell DNA PCR amplification and provides improved discrimination between live and dead bacteria [136]. Furthermore, combining immunomagnetic separation (IMS)—which uses specific antibodies to capture and concentrate target pathogens from complex samples like PIF—with PMAxx-ddPCR has been shown to achieve high accuracy and sensitivity [137]. For example, targeting the internal transcribed spacer (ITS) region of *C. sakazakii*, this IMS-PMAxx-ddPCR approach enabled detection of VBNC *C. sakazakii* in PIF at levels as low as 5.6 copies/g [138].

Recent innovations in microfluidic chip technology, which miniaturizes and integrates entire nucleic acid detection workflows onto a single device, are gaining traction for their portability, low reagent consumption, and operational simplicity across multiple fields [139,140,141,142]. Tang et al. [143] created an automated, fully enclosed microfluidic system that couples magnetic bead-based extraction with LAMP for low-cost, hands-off detection. Meanwhile, biosensor platforms combining aptamers or nanobodies with nanomaterials—such as gold nanoparticles—enable label-free, visual, and quantitative detection within 20 min at sensitivities as low as 10 CFU/mL [113]. These emerging systems offer the advantages of portability and negligible cross-reactivity but still face challenges in standardization and robustness in complex food matrices.

### 4.2. Identification of C. sakazakii

Once *C. sakazakii* is isolated from samples, accurate identification of the isolate is crucial for confirming diagnosis, tracing outbreaks, and assessing pathogenic potential. Traditional biochemical identification methods are time-consuming and often lack resolution for discriminating closely related *Cronobacter* species [144].

16S rRNA gene sequencing serves as a definitive tool for confirming the genus and resolving ambiguous results by comparing sequences to reference databases [145]. However, species-level identification remains a challenge. Analysis based on the 16S rRNA gene fails to differentiate between *C. sakazakii* and *C. malonaticus*, as these two species are phylogenetically very close [146]. Recently, genomics-driven target screening has identified new markers, including *yifL* and *fimG*, that may improve assay specificity and support more reliable species discrimination [7].

Matrix-assisted laser desorption/ionization time-of-flight mass spectrometry (MALDI-TOF MS) permits high-throughput identification of *C. sakazakii* within minutes and is increasingly supplanting traditional techniques in clinical settings [147,148]. The identification process requires a pure colony, which is directly applied to a target plate with matrix; spectra are compared against a reference database. This approach drastically reduces turnaround time compared to biochemical identification [149]. At present, conventional molecular biology and mass spectrometry techniques are difficult to stably distinguish between closely related species within a genus and highly virulent subspecies due to limited taxonomic resolution, protocol sensitivity, or database dependence, resulting in false positives, failed species-level identification, or misjudgment of pathogenicity in detection results [150]. In addition, existing methods generally lack the ability to distinguish between live and dead bacteria, and overly rely on pure culture or standardized library conditions, resulting in technical blind spots for accurate qualitative and quantitative analysis in complex matrices (such as PIF) [151]. It is urgent to combine high-throughput sequencing with novel biomarker mining to fill this gap.

WGS, the current gold standard for strain typing and outbreak tracing, provides unparalleled resolution by distinguishing hypervirulent clones (e.g., ST1, ST4) through core genome multilocus sequence typing, while also predicting the distribution of pathogenicity islands and antibiotic resistance determinants [152]. Despite its comprehensive nature, WGS remains limited by high cost and analytical complexity.

## 5. Infection Control and Prevention

**Figure 2:**
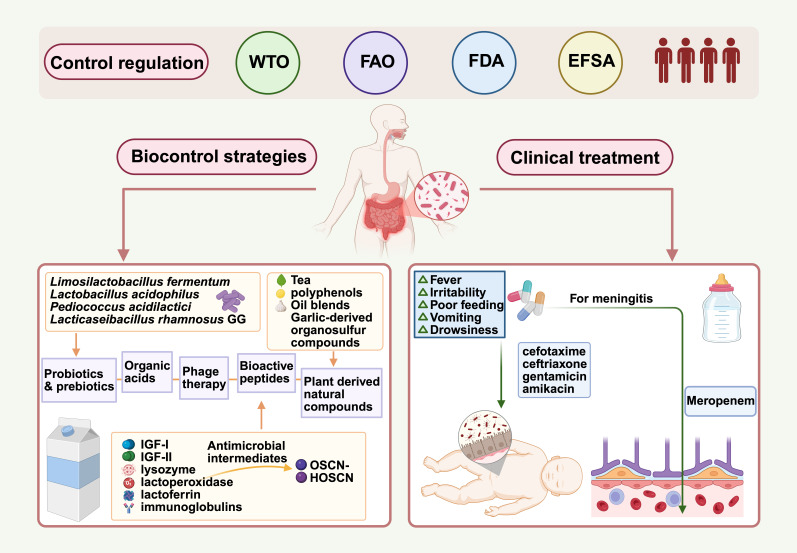
Prevention and control strategies for *C. sakazakii*. Major international regulatory agencies (WTO, FAO, FDA, EFSA) have established control guidelines for *C. sakazakii* in the food industry. Advanced biocontrol approaches have been explored, such as the use of probiotics and prebiotics, bioactive peptides, organic acids, bacteriophage therapy, and plant-derived natural compounds. For clinical management, treat with antibiotics effective based on susceptibility testing, prioritizing those with good Blood–Brain Barrier penetration (e.g., meropenem) for suspected meningitis. Created in BioRender. Zhang, C. (2026) https://BioRender.com/zo6xp1m (accessed on 21 February 2026).

Given the severity of *C. sakazakii* infections, effective control and prevention strategies are paramount. These efforts span from international regulatory frameworks and clinical therapeutics to novel biocontrol approaches, as summarized in Figure 2.

### 5.1. Regulation

Due to the great public health significance of *C. sakazakii*, international organizations and national authorities have established specific regulatory frameworks to minimize contamination in PIF. The European Food Safety Authority (EFSA) [153] provides guidance on preventing microbiological risks in infant formula, such as using sanitized containers both in households and hospitals. In the United States, following a major *C. sakazakii* contamination incident, the Food and Drug Administration (FDA) implemented an organizational restructuring to strengthen oversight of critical foods, including both production environments and finished products to better control such pathogens [154]. At the international level, the World Health Organization [155] highlights concern over *C. sakazakii* in PIF, provides risk assessments, and offers recommendations for safer practices. Among these, the Food and Agriculture Organization of the United Nations (FAO) and the WHO [156] jointly advise reconstituting infant formula with water at a temperature above 70 °C to reduce or eliminate the potential risk of *C. sakazakii* contamination.

### 5.2. Therapeutics

*C. sakazakii* infections progress rapidly, often leading to an unfavorable prognosis and even death. Given this rapid progression, early recognition remains critical in clinical practice, particularly among high-risk populations. Newborns, premature or low-birth-weight infants, as well as immunocompromised infants who present with symptoms such as fever, irritability, poor feeding, vomiting, or drowsiness, and have a history of PIF intake, should be promptly evaluated for *C. sakazakii* infection [157]. Once infection is confirmed, treatment becomes a major concern. Currently, there is no standard domestic or international consensus guideline specifically for the management of *C. sakazakii* infections. Antibiotic therapy remains the mainstay of treatment. Traditionally, a combination of ampicillin with gentamicin or chloramphenicol was recommended, but increasing resistance to these antibiotics has been reported in recent years [27,158]. More recently, a third-generation cephalosporin (e.g., cefotaxime or ceftriaxone) combined with an aminoglycoside (e.g., gentamicin or amikacin) has been recommended, with therapy adjusted based on antimicrobial susceptibility results [5].

Given the organism’s propensity to cause meningitis and brain abscesses, CSF testing and neuroimaging are important for guiding management [159]. The ability of *C. sakazakii* to utilize immature dendritic cells and survive inside human macrophages suggests that it has immune-evasion mechanisms allowing it to escape host defenses and subsequently access and cross the BBB [35]. Therefore, drugs with the ability to penetrate the BBB, such as meropenem, are crucial for treating meningitis and are recommended for critically ill patients [11,160,161,162].

### 5.3. Biocontrol Strategies

#### 5.3.1. Probiotics, Prebiotics, and Synbiotics

Probiotics and prebiotics have been widely investigated for modulating the naïve gut microbiota, while synbiotics—a combination of both—are increasingly recommended to harness their synergistic effects [163,164]. Pre-colonization of the intestinal mucus layer by probiotic strains can reduce the adhesion and subsequent infection of *C. sakazakii* [165]. For instance, co-culture studies demonstrated that *Limosilactobacillus fermentum*, *Lactobacillus acidophilus*, and *Pediococcus acidilactici* reduced viable *C. sakazakii* counts by 50% during five minutes of contact [166]. Additionally, *Lacticaseibacillus rhamnosus* GG has been shown to be an effective probiotic that can inhibit *C. sakazakii* adherence and promote intestinal health [167].

Prebiotics likewise appear to interfere with pathogen colonization. Polydextrose and galacto-oligosaccharides have been reported to inhibit *C. sakazakii* adhesion to HEp-2 cells during the initial stages of infection, either individually or in combination [168]. Mixed microbial consortia may offer even greater protective effects [169]. For example, Kefir supernatant was found to completely inhibit the growth of *C. sakazakii* when added to reconstituted milk formula within one hour at 30–50% concentration [170]. Such findings highlight their potential role in providing clinical protection against this pathogen.

#### 5.3.2. Bioactive Peptides

Bovine colostrum is reported to contain many bioactive components such as insulin-like growth factors I and II (IGF-I and IGF-II), lysozyme, lactoperoxidase, lactoferrin, and immunoglobulins [171]. These bioactive substances exhibit an inhibitory effect on the intestinal adhesion of *C. sakazakii* [172]. Besides these milk components, activation of the lactoperoxidase system generates two antimicrobial intermediates: hypothiocyanite ion (OSCN^-^) and hypothiocyanous acid (HOSCN). Both compounds exhibit inherent antimicrobial properties and exert their antimicrobial activity by oxidizing the sulfhydryl groups on enzymes and cytoplasmic membrane proteins, leading to the disruption of essential cellular functions [173].

#### 5.3.3. Phage Therapy

Phage therapy can effectively address the issue of antibiotic resistance in foodborne pathogens, including *C. sakazakii*. A Swiss study has shown that high concentrations (10^9^ PFU/mL) of *C. sakazakii*-specific bacteriophages completely prevented bacterial growth across a range of temperatures [174]. Because of their inherent strain specificity, single phages have limited coverage; however, phage cocktails can substantially broaden host range and enhance efficacy. A five-phage cocktail eliminated 35 of 40 tested *C. sakazakii* strains in artificially contaminated PIF [175], while another formulation (phages leB, leE, leN) inhibited 73% of strains in different PIF brands. Notably, a 3 × 10^8^ PFU/mL cocktail reduced bacterial counts below the detection limit (10 CFU/mL) and suppressed biofilm formation [176]. The high specificity of bacteriophages, combined with their inability to infect eukaryotic cells, makes them attractive candidates for ensuring food safety. However, they may trigger humoral immune responses, and further long-term safety studies are needed before large-scale application in infant nutrition systems.

#### 5.3.4. Organic Acids

Several organic acids have demonstrated antibacterial activity against *C. sakazakii*. Screening of 51 isolates proved propionic acid (MIC 16–31 mM) and acetic acid (MIC 31–63 mM) as the most potent in liquid media [177]. Phenolic acids such as lipoic acid, syringic acid, and ferulic acid also exhibit antibacterial activity, with MIC values ranging from 2.5 to 5.0 mg/mL, likely attributable to their inherent functional groups and lipophilic properties. Mechanistic studies suggest that these acids compromise membrane integrity through intracellular ATP depletion, changes in cytoplasmic pH, and induction of cell membrane hyperpolarization [178,179].

#### 5.3.5. Plant-Derived Natural Compounds

Polyphenols and other plant-derived compounds have been proposed as potent anti-*C. sakazakii* agents due to their minimal or no adverse effects, wide availability, and multifaceted mechanisms of action [180]. Tea polyphenols (TP) were able to reduce *C. sakazakii* counts by up to 7 log CFU/mL within 1 h with enhanced bactericidal efficacy in rehydrated PIF acidified to pH 3.5. Mechanistic analyses indicated that TP exerted an irreversible bactericidal effect, mainly through the disruption of outer and inner bacterial membranes, ultimately leading to cytoplasmic leakage and cell death [181]. Other natural antimicrobials have also shown promising effects against *C. sakazakii*. Essential oil blends, such as fir and cinnamon oils, rapidly reduced *C. sakazakii* populations in reconstituted PIF to undetectable levels within 3 h [182]; while garlic-derived organosulfur compounds (diallyl sulfide and Z-ajoene) achieved similar eradication within 8 h in a dose-dependent manner. Coenzyme Q_0_ from *Antrodia cinnamomea* exhibited potent anti-*C. sakazakii* activity (MIC 0.1 mg/mL) and significantly reduced biofilm formation at 4 mg/mL [183].

#### 5.3.6. Practical Challenges and Research Gaps

Despite the promise of these biocontrol approaches, several challenges must be addressed before their widespread implementation, particularly in sensitive populations such as infants. Regulatory frameworks for novel interventions—especially bacteriophages and probiotics—remain fragmented and inconsistent across jurisdictions [184,185]. For instance, the classification of bacteriophages as either food additives or processing aids varies between regulatory bodies, creating uncertainty for commercial applications [186]. Stability presents another critical hurdle: many biocontrol agents, including plant-derived compounds and probiotics, are susceptible to degradation under gastrointestinal conditions or during food processing and storage, necessitating advanced formulation strategies such as electrospinning and electrospraying, or microencapsulation to ensure efficacy [187,188,189]. Safety considerations also extend to potential off-target effects, metabolite toxicity, and the risk of horizontal gene transfer in the case of phages [190,191]. Importantly, the majority of current evidence derives from in vitro assays or food-model systems, with a notable paucity of well-controlled clinical trials [192]. Bridging this translational gap through rigorous human studies will be essential to validate both the efficacy and safety of biocontrol strategies in real-world settings and to support their regulatory acceptance.

## 6. Conclusions

*C. sakazakii* remains a formidable opportunistic pathogen of significant concern in neonatal health due to its environmental resilience, diverse virulence arsenal, and capacity to survive in low-moisture foods [193,194,195,196]. *C. sakazakii* employs multiple strategies to invade host tissues and establish infection [88]. Following mucosal contact, *C. sakazakii* employs a classic infection strategy: it begins with mucosal colonization, then evades host defenses by invading cells or surviving phagocytosis, subsequently spreading systemically via the bloodstream to sites like the meninges, and ultimately causing host damage through toxins or inflammatory responses [197]. In addition, *C. sakazakii* demonstrates remarkable environmental persistence, especially in low-moisture foods such as PIF, with a desiccation tolerance exceeding that of other foodborne pathogens, including *Salmonella*, *Listeria monocytogenes*, and *E. coli* [18,198]. Although significant progress has been made in elucidating its molecular pathogenesis, biofilm formation, and stress response systems, major challenges persist in early diagnosis, effective disinfection, and targeted therapeutics.

Future efforts should focus on developing rapid, field-deployable diagnostic platforms that enable early and precise identification of *C. sakazakii* in food and clinical samples, including technologies integrating microfluidics, biosensors, and machine learning-driven analytics. In parallel, innovative developments in genomics and surveillance should be expanded to track emerging hypervirulent or drug-resistant strains more precisely.

Preventive strategies must also evolve beyond conventional hygiene practices. The integration of biocontrol measures—such as bacteriophages, probiotics, and plant-derived antimicrobials—provides promise for reducing contamination along the “farm-to-table” chain. In clinical settings, the treatment of *C. sakazakii* faces severe challenges, with the core issue being its increasingly severe multidrug resistance. Many strains exhibit high-level resistance to first-line antibiotics, which limits or even renders traditional treatment methods ineffective. At the same time, the bacterium’s strong environmental survival ability and complex virulence mechanism further increase the difficulty of clinical prevention, control, and eradication. Novel therapeutic regimens that enhance blood–brain barrier penetration and limit intracellular persistence warrant further exploration. Effective control of *C. sakazakii* will ultimately require a One Health framework that integrates food, clinical, and environmental monitoring to ensure comprehensive public health protection.

## Figures and Tables

**Table 1:** Comparison of methods for the detection of *C. sakazakii*.

Method	Principle	Pre-Detection Steps	Pre-Treatment Time	Assay Time	Limit of Detection	Reference
Traditional culture	Cultural isolation	Pre-enrichment; selective enrichment	40–44 h	24 h	Qualitative	[108]
ELISA	Antigen–antibody detection	Mix; enrichment; centrifugation	4.5 h	3.5–4 h	1 × 10^4^ cfu/mL (pure culture); 1 cfu/g (in PIF after enrichment)	[109]
Conventional PCR	Nucleic acid amplification	Enrichment; boiling; centrifugation	6.5 h	2–2.5 h (including agarose gel separation and visualization)	1.41 pg/PCR	[110]
qPCR	Nucleic acid amplification	Enrichment; DNA extraction	12.5 h	1.5–2 h	3.44 log CFU/mL (g) (without enrichment); 0.03 log CFU/mL (after 12 h enrichment)	[111]
LAMP	Nucleic acid amplification	DNA extraction	<30 min	1 h	9.1 fg/μL (DNA); 10^1^ CFU/mL (sample)	[112]
Biosensors	Nanobody-induced AuNP aggregation	Dissolution; centrifugation; dilution; filtration	10–15 min	20 min	136 CFU/mL (quantitative); 10^3^ CFU/mL (visual)	[113]

**Table 2:** Comparison of methods for the identification of *C. sakazakii*.

Method	Principle	Pre-Identification Steps	Preparation Time	Identification Time	Limit of Identification	Reference
Traditional biochemical identification	Biochemical reaction	Isolation; purification	48 h	<10 h	0.5–0.63 McFarland	[108]
16S rRNA gene sequencing	Nucleic acid amplification	Strain cultivation; DNA extraction	1–2 d	2–2.5 h	10 pg/PCR	[114]
MALDI-TOF MS	Protein fingerprinting	Pre-enrichment; cell collection and washing; chemical and physical lysis; target plate spotting and drying	6.5 h	0.4 min	4.1 × 10^1^ cfu/mL (PBS); 2.72 × 10^3^ cfu/mL (PIF)	[115]
WGS	Genome sequencing	Strain resuscitation and purification; DNA extraction; library construction	3–4 d	2–3 d	Single colony	[116]

## Data Availability

No new data were created or analyzed in this study. Data sharing is not applicable to this article.
